# Experimental Investigation of Bi-Directional Flax with Ramie Fibre-Reinforced Phenol-Formaldehyde Hybrid Composites

**DOI:** 10.3390/polym14224887

**Published:** 2022-11-12

**Authors:** Durvasulu Rajesh, Nagarajan Lenin, Robert Cep, Palanivel Anand, Muniyandy Elangovan

**Affiliations:** 1 Department of Mechanical Engineering, Vel Tech Rangarajan Dr. Sagunthala R&D Institute of Science and Technology, Avadi 600 062, India; 2 Department of Machining, Assembly and Engineering Metrology, Faculty of Mechanical Engineering, VSB-Technical University of Ostrava, 17. Listopadu 2172/15, 708 00 Ostrava, Czech Republic; 3 Department of R&D, Bond Marine Consultancy, London EC1V 2NX, UK

**Keywords:** flax, ramie, phenol formaldehyde resin, vacuum infusion process, mechanical testing, scanning electron microscope

## Abstract

Modern research focuses on natural, green, and sustainable materials that can be used to replace conventional materials. Because of their beneficial qualities, natural fibre composites are being thoroughly researched. This research focuses on the development of a flax fibre reinforced with phenol-formaldehyde resin hybridization with ramie fibre through a vacuum infusion process. Eight different sequences were fabricated using a core–sheath structure and were mechanically characterized as per ASTM standards. The fabrication technique influences the adhesion of the matrix with reinforcement. The results also reveal that composite having ramie as a sheath layer and flax as a core delivers good mechanical characteristics compared to vice versa. The laminate H exhibited highest mechanical properties among all the eight laminates produced for this study. It exhibited a tensile strength of 54 MPa, tensile modulus of 0.98 Gpa, elongation of 7.1%, flexural strength of 143 Mpa, and compressive strength of 63.65 Mpa. The stress strain curves revealed that all the laminates exhibited ductile behaviour before failing during the tensile test and flexural test, respectively. The stacking sequence of the laminate H influenced the mechanical properties exhibited by it and its counterparts. A morphological study was carried out to analyse the failure surfaces. Morphological analysis exhibited few defects in the laminate after the tests. The composites developed delivers better mechanical properties than commercial composites available on the market, which can be used in lightweight structural applications.

## 1. Introduction

In past few years, there has been an increase in the use of synthetic fibres (e.g., carbon and glass) for strengthening or reinforcing engineering structures [1,2,3]. with regard to this application, the fire resistance performance of synthetic fibres is a significant challenge and declines abnormally when exposed to elevated temperature [4,5,6]. A potential alternative is the hybridization of a composite made of polymeric matrices reinforced with synthetic and natural fibres, which gives good mechanical enactment [7,8,9]. Natural fibres have lower specific gravity and are biodegradable when compared to synthetic fibres [10]. However, natural fibres frequently exhibit difficulty in compatibility among fibre and polymer matrixes. It is caused by the hydrophobic nature of fibre and matrix incompatibility, which can also be improved by surface modification [11,12,13]. Natural fibres, particularly bast fibres, are an excellent substitute for conventional fibres due to their ease of extraction, availability, light weight, low density, biodegradability, and high specific strength [14,15,16]. Natural fibre qualities vary depending on how they are grown and extracted [17,18,19]. To replace the application of synthetic fibres in various fields such as aerospace, automotive growth of natural fibre-based composites is widely considered. Hybridization represents an evolution for widening the function of composite materials, especially in advanced applications, by maximizing toughness or impact resistance [20]. Naveen et al. studied the mechanical properties of Kevlar/Cocos nucifera sheath-reinforced composite with different weight fraction and reported that Cocos nucifera sheath has the potential to replace the Kevlar fibre polymer composite [21]. Giridharan assessed the characteristics of a glass/ramie fibre-reinforced composite at various weight percentages. The addition of a small fraction of glass to the fibre resulted in increased properties of ramie, making it low-cost and eco-friendly [22]. Yang et al. concentrated on the effects of unmodified ramie fibre-reinforced polypropylene using melting hybrid technology to acquire good mechanical properties. Modified fibre thereby has more fibre reinforcement than unmodified ramie fibre. The thermal degradation temperature is reduced because of the polypropylene/ramie fibre [23]. Composite laminate fabrication is shifting away from traditional hand layup processes and toward new techniques such as resin infusion, vacuum-assisted resin transfer moulding (VARTM), vacuum bagging, etc. It results from lower labour, material, and equipment costs, which increase the quality and affordability of producing parts. Natural fibre-reinforced composite laminate made using bio-based epoxy resin offers a high fibre volume fraction and low processing costs. The developed composite is intended for industries that require cheap cost, light weight, and a minimal carbon footprint [24,25,26,27]. Sanjeevi et al. used the hand layup method to investigate the effect of a hybrid natural fibre phenol–formaldehyde composite. Three different weight percentages were considered (25%, 35%, 45%). The 35% fibre reinforcement composite performed better in fibre–matrix bonding than the other two fabricated composites [28]. Grafting of nano-silica to surface of ramie fibre improves the surface roughness, which results in the enhancement of mechanical and thermal properties [29]. A shear lag model was developed to study the load transfer mechanism of composites in nanohybrid shish-kebab structures. It shows pronounced effects in elastic modulus and morphology [30]. Swamy et al. investigated the effect of areca fibre on its strength. Areca-treated phenol-formaldehyde absorbs a significant quantity of moisture early on, and biodegradation occurs slowly, which is advantageous in structural applications and packaging sectors [31]. The moisture, chemical resistance, and mechanical properties of pine needles are reinforced with phenol–formaldehyde, which is essential in selecting applications in various fields [32]. Joseph et al. observed that banana fibre, when soaked with phenol–formaldehyde, improves mechanical characteristics and interfacial shear strength when compared to glass fibres [33]. Sathyaseelan et al. studied the influence of stacking sequence in the hybrid composite, which is essential to increasing the mechanical properties of composite laminate [34]. Because of rising environmental awareness and its great quality to substitute fossil fuel and non-renewable resources in reinforcing composite materials, many researchers have examined the sustainability of natural fibre in diverse applications [35,36]. Hybridization is the process of combining two or more elements to create a composite that can be totally natural, completely synthetic, or a blend of natural and synthetic materials. Weight fraction, stacking sequence, volume fraction, chemical treatment, and ambient variables are all important elements in composites [37,38,39,40].

As per the reviewed literature, there has been little study on the mechanical evaluation of thermoset-based hybrid composite laminates made using flax/ramie/phenol–formaldehyde constituent materials. The hybridization method was employed to generate low-cost composites. The main objective of this study is to investigate the influence of the stacking sequence of flax/ramie fibre-based hybrid composites on their mechanical properties, such as tensile, flexural, compressive, impact, and hardness. Morphological analysis was conducted using a scanning electron microscope to study the fractured surface of the composite.

## 2. Materials and Methods

### 2.1. Flax Fibre

**Figure 1:**
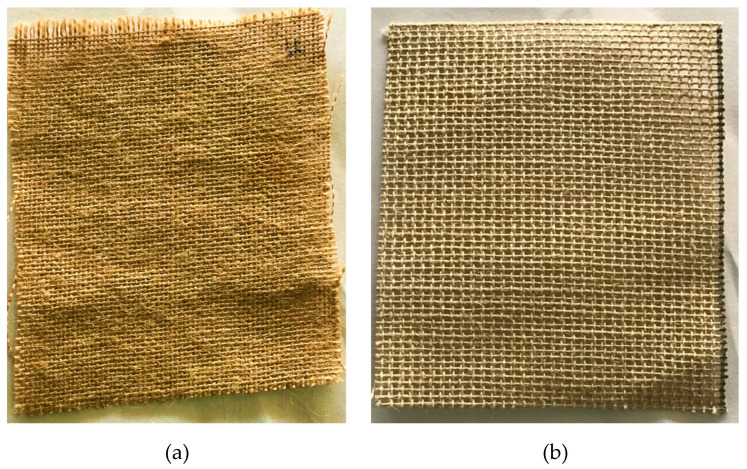
Bidirectional woven sheet of (**a**) flax fibre (**b**) ramie fibre.

Flax fibre is derived from flowering plants in the *Linum Usitatissimum* species, as illustrated in Figure 1a. Flax fibre, extracted from the plant, is moderately stronger than cotton fibre. Because it is a robust and sturdy fibre, it is mostly employed in the textile industry in Western countries. The benefits of flax fibre include its density, renewable nature, reduced hazard as compared to glass fibres, and the fact that objects made from flax do not tend to lose their shape. Fibres were purchased from Go Green Products Pvt. Ltd., Chennai, India.

### 2.2. Ramie Fibre

Ramie fibre is generated from a flowering plant of the *Urticaceae* family, as seen in Figure 1b. It is one of the strongest fibres and retains its strength when wet. It is used in the packaging industry, fishing nets, and to a lesser extent in clothing and fabrics. The physical properties of the material used for fabrication are presented in Table 1.

### 2.3. Phenol–Formaldehyde

Because of its superior surface smoothness, strength, low cost, and high fire resistance, phenol–formaldehyde, often known as phenolic resin, is gaining popularity over other resins. These resins are synthetic polymers made by reacting phenol with formaldehyde. Phenol–formaldehyde resin is primarily used in production of circuit boards. The resin is mixed with a hardener in a ratio of 12.5:1. Resin and hardener are stirred continuously for 5 min to apply the mixed catalyst for 30 min. Curing and post curing of composites is commended to obtain optimal mechanical properties. The fabricated composite laminate is cured at room temperature. ABR Organics Limited Telangana (Hyderabad, India) supplies phenol–formaldehyde resins and the hardener.

### 2.4. Vacuum Infusion Process

**Figure 2:**
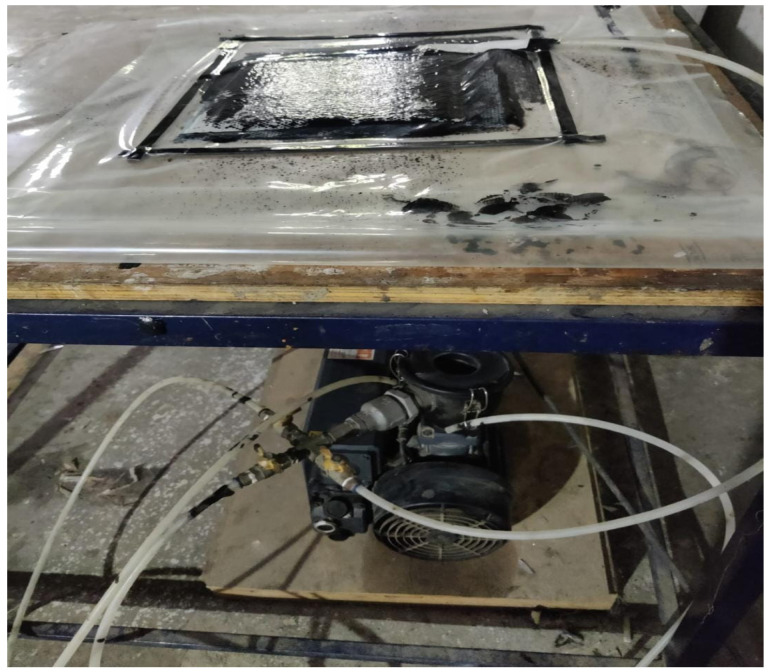
Vacuum infusion process.

The current study employed the vacuum infusion process to fabricate composite laminate. Manufacturing high-strength composite parts comparable to composite laminates created from prepreg, the autoclave process, and so on is cost-effective. In most cases, the vacuum infusion process is carried out in a closed system. A perforated film is placed in the vacuum bag during this process; dry fibre and release film are placed on top of the mould surface and sealed inside the vacuum bag [41]. Vacuum bag setup was constructed in house. Figure 2 illustrates how the vacuum force moves resin down a symmetry line from the resin container into the vacuum bag. The presence of more fibre causes the impregnation time to be delayed.

### 2.5. Composite Specimen Preparation

**Figure 3:**
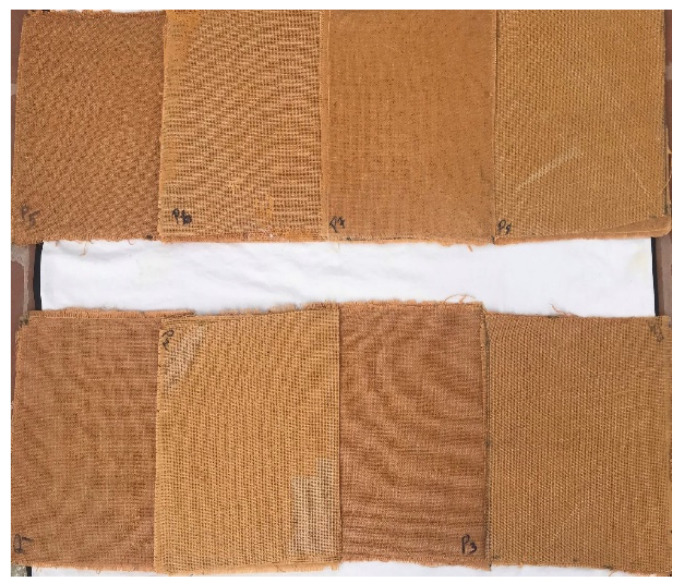
Fabricated laminate.

The fabrication of composite specimens was prepared by vacuum infusion process. It is one of the most cost-effective manufacturing techniques among moulding techniques. Phenol–formaldehyde resin along the hardener was mixed and used as a matrix in a ratio of 12.5:1. The bottom of the mould was coated with releasing agent for easy removal of the specimen, and the first layer of fibre was kept over the coated surface after drying of releasing agent. Subsequently, the other four layers of fibres were kept one after the other and the resin was driven through the laminate using vacuum pressure. Once a complete vacuum was achieved the resin was sucked into laminate via the careful placing of the tube. Eight different stacking sequences were used in this experimental study as shown in Figure 3. The fabricated composite laminate was a size of 300 × 300 × t mm^3^. It was allowed to cure for 24 h at room temperature. Once the curing process was completed, the laminate was removed from the vacuum setup and cut according to ASTM standards. The stacking sequence of fabricated hybrid laminate and its configuration are shown in Table 2.

### 2.6. Composite Characterization

The tensile test was performed in the FMI Universal Testing Machine (UTM) (Perfect Enterprises, New Delhi, India) following the ASTM: D638 standard, with dimensions of 165 mm × 19 mm and a crosshead speed of 2.5 mm/min. Tensile strength is important in determining a material’s ability to bear a load when exposed to tension in a UTM. Because both the reinforcement and the matrix material were brittle, the final composite material was also fragile. The flexural specimens were made by ASTM D790 standards, with 127 mm × 12.7 mm dimensions and a crosshead speed of 2.5 mm/min. The three-point bending test was used for composite flexural testing, and the load was applied under precise conditions. The gauge at the bottom of the specimen was used to measure the deflection. The Izod impact test was carried out using Impact testing machine model XJJU 5 under ASTM standard D256, with dimensions of 65.5 mm × 12.7 mm. The machine is made up of a loading striker that has fixed kinetic energy when released. The dial indicates the amount of energy absorbed. The compression test was carried out in an ASTM: D695-compliant universal testing machine made by FMI, with dimensions of 70 mm × 19 mm. It governs the material’s behaviour when the specimen is crushed under load. Finally, the Shore D gadget performed the hardness test by ASTM standard D2240. It measures the depth of an indentation in a material by applying the needed force in a constant matter without shock with a standard presser foot. The morphological analysis of developed composites was examined by scanning electron microscopy. F E I Quanta FEG 200 machine was used to capture the image. Gold sputtering of samples was performed to improve conductivity before the microstructure study was carried out in polymeric-based specimens. To evaluate the mechanical properties of the composite laminate a minimum of three samples were prepared, and the average of the three values was taken for discussion.

## 3. Results and Discussion

### 3.1. Tensile Strength

**Figure 4:**
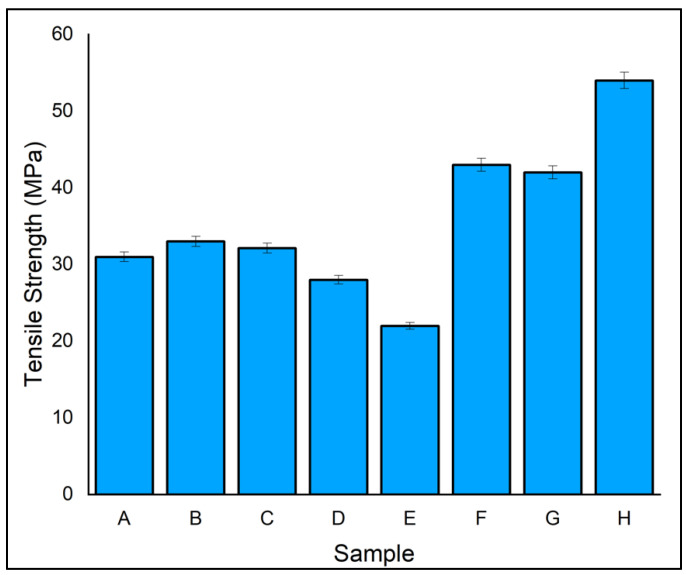
Tensile strength of specimen.

Figure 4 depicts the tensile strength of eight different fabricated laminates. The specimens fractured between the tensile grips and at the gauge region; such phenomena occur during tensile testing under constant stress conditions arising at the gauge region. Furthermore, the entire specimen failed in a brittle manner in all cases, and the same can be seen in stress–strain behaviour. Among all the eight laminates, the highest strength of 54 MPa was exhibited by laminate H, which had an outer layer of ramie fibre and core of flax fibre due to good bonding to the matrix and reinforcement of the vacuum infusion process. On the other hand, hybrid laminates D and F, which had an outer and alternate layer of ramie fibre, exhibited a tensile strength of 48.16% and 20.39% lower tensile strength than laminate H, respectively. laminate E, which consisted of an alternating layer of flax and ramie fibres, exhibited a lower tensile strength of 21.99 MPa. On the other hand, laminates A and B showed 38.89% and 42.61% lower tensile strength than laminate H, respectively. Furthermore, the maximum tensile strength of the banyan/ramie fibre-reinforced hybrid composite dealt by Raja et al. was 24.63% lower compared to laminate H since they fabricated the laminates using the hand layup technique [42]. This shows the influence of fabrication technique also plays a pivotal role in hybrid composites. Similarly, the result of Mohanavel et al. for the jute and ramie fibre combination was 35.18% lower compared to the tensile strength of laminate H [43]. Chemical compositions such as cellulose, wax content, and fibre angle are controlled by the tensile properties of natural fibre [44]. Hence, upon seeing the tensile strength of hybrid laminates, it was concluded that reinforcement and hybridization have a positive impact on the tensile properties.

**Figure 5:**
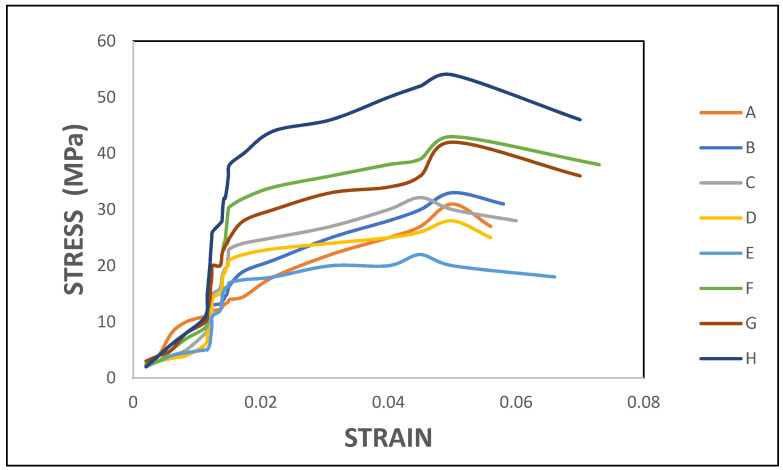
Stress vs. strain curve during tensile testing.

Figure 5 depicts the stress vs. strain relation of eight different fabricated laminates. The graph was plotted from the result obtained during the tensile test. It is shown that all the eight laminates have a sudden raise in the stress value when the strain was around 0.013. Until this point, the stress varied at different rates respective to the composition and stacking sequences of the laminates. It is inferred that the stacked layers of the reinforcement materials offered resistance against the external load. Laminate B maintained an almost constant stress of 20 MPa for the majority of the strain. However, this laminate attained a maximum stress of 23 MPa at the 0.044 strain. Compared among the eight laminates, this was the lowest. It is inferred that the five layers of ramie fibres were able to absorb relatively less load causing it to exhibit minimal stress. However, the strain value was considerably greater than in laminates such as A, C, D, and E.

Among all the eight laminates, the highest stress of 54 MPa was exhibited by laminate H, which had an outer layer of ramie fibre and core of flax fibre due to good bonding of the matrix and reinforcement by vacuum infusion process. This hybrid laminate continued to absorb the load, gradually increasing its stress until it attained the value mentioned above. After that, the stress decreased slowly before it failed at a strain value of 0.07. The trend in the stress-strain curve shows that this hybrid laminate exhibited ductile behaviour. Furthermore, all the test specimens failed in a ductile manner in all cases.

**Figure 6:**
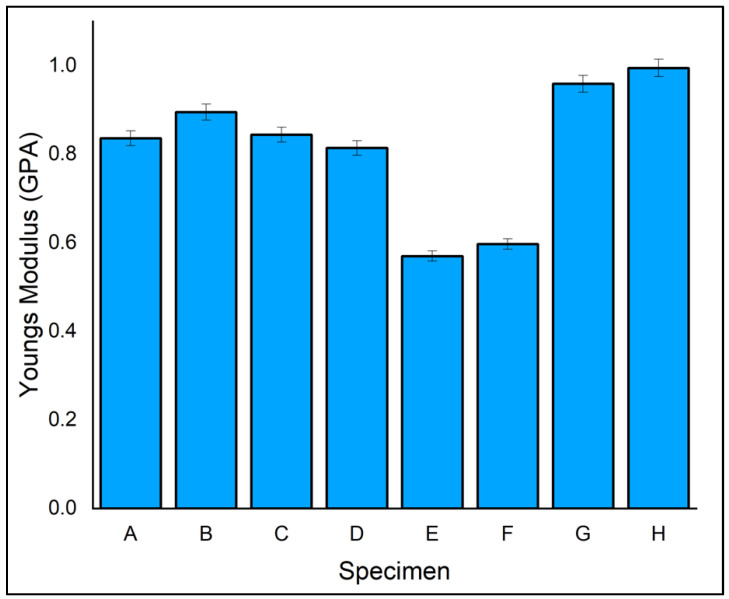
Young’s modulus of the composite.

Figure 6 depicts the tensile modulus relation of eight different fabricated laminates. The error bar is plotted along in the bar chart plotted for the tensile modulus. These error bars were obtained by taking three tests continuously for the same laminate material. Among all eight laminates, the highest tensile modulus of 0.98 GPa was exhibited by laminate H, which had an outer layer of ramie fibre and core of flax fibre due to good bonding of matrix and reinforcement by vacuum infusion process. Laminate G showed the second-highest tensile modulus of 0.96 GPa, which had an outer layer of flax fibre and ramie fibre as core due to good bonding of matrix and reinforcement by vacuum infusion process. The trend was similar to the result obtained for tensile strength. This reveals that the stacking sequence of the reinforcements plays a vital role in their properties [45]. It is inferred that sandwiching three consecutive layers of the same fibres potentially increases the tensile moduli of the laminates.

**Figure 7:**
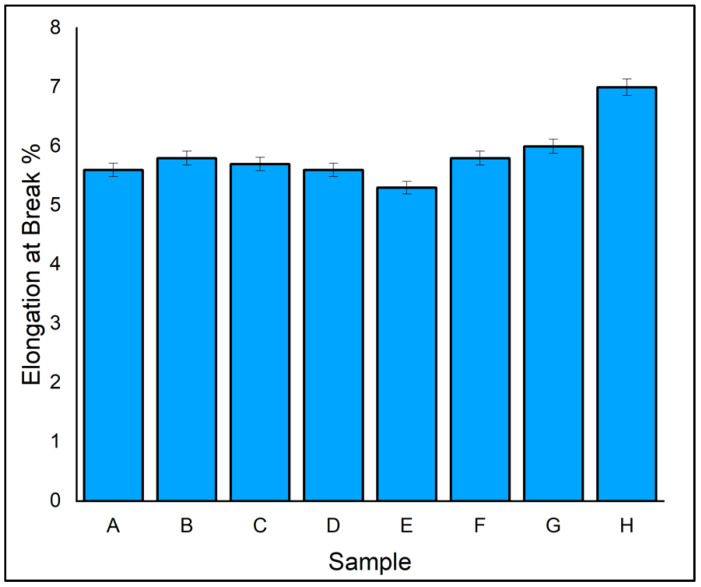
Elongation of break (%) of composite.

Figure 7 depicts the elongation relation of eight different fabricated laminates. All the composites had more than 5% elongation. This reveals the ductile behaviour of the laminates. Among all eight laminates, the highest elongation of 7.1% was exhibited by laminate H, which had an outer layer of ramie fibre and core of flax fibre due to good bonding of matrix and reinforcement by vacuum infusion process. The hybrid laminates D and F, which had an outer and alternate layer of ramie fibre, exhibited elongation of 1.6% and 1.8% lower than laminate H, respectively. This shows that the influence of fabrication technique also plays a pivotal role in hybrid composites. It is concluded that reinforcement and hybridization have a positive impact on elongation properties.

### 3.2. Flexural Strength

**Figure 8:**
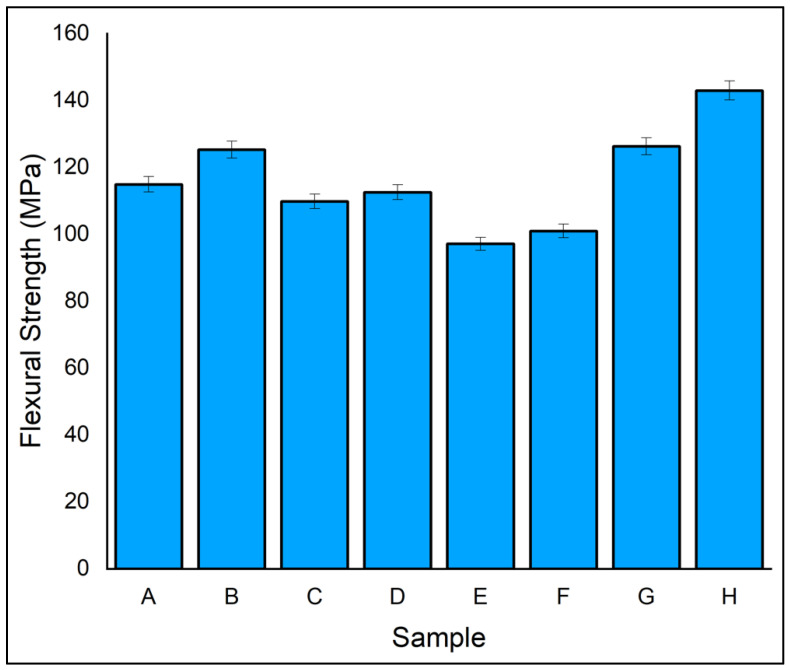
Flexural strength of specimen.

The material flexibility plays a critical role in resisting the loads when it acts perpendicular to the laminate plane. The flexural behaviour of the fabricated laminates is brittle, as is the tensile behaviour. In the present study, a three-point flexural analysis was used to find the flexural strength of laminates and the results are compared in Figure 8. A maximum flexural strength of 143 MPa was obtained for laminate H and a minimum flexural strength of 97.15 MPa for laminate E. The flexural strength of laminate B, which had all five layers of ramie fibre, was 125.3 MPa, which was 8.26% more than its counterpart laminate A. The results show that the flexural strength of the hybrid laminates was maximum when the laminate had ramie as the outer layer rather than flax. Furthermore, it is inferred that the hybridization of ramie with flax fibre had significantly enhanced the flexural properties of the laminates due to fibre hybridization. The sequence of fibres influenced the flexural characteristics of the composite. As ramie has good flexural strength, the same was reflected in ramie as shell and flax as the core laminate [46,47]. Similarly, ramie and flax placed as alternative layers resulted in better flexural strength than other laminates. When flexural loads are applied to a composite specimen, the fibres at the external position are highly stressed [48]. Proper penetration of resin into fibre in flexural strength is highly dependent on adhesive properties of fibre and matrix [49]. It has additionally been stated that the hybridization of natural fibre provides better flexural strength than single fibres [50].

**Figure 9:**
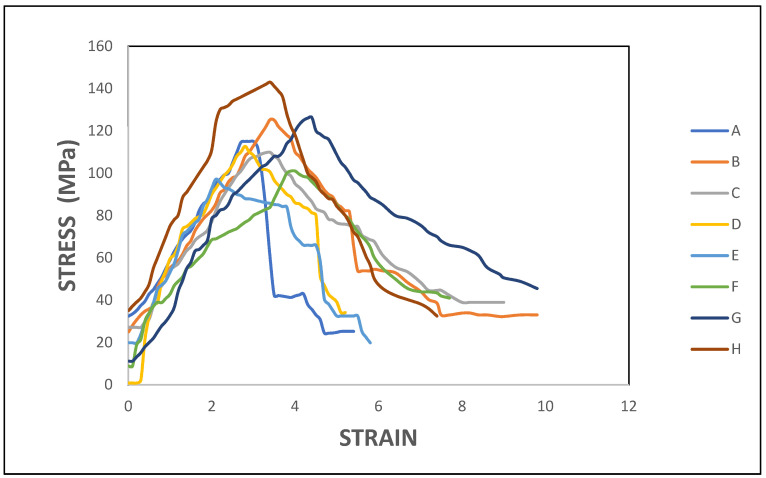
Flexural stress vs. strain for composite.

Figure 9 depicts the flexural stress vs. strain relation of eight different fabricated laminates. The graph was plotted from the results obtained during the flexural test. All the laminates exhibited a similar trend in the changes to stress and strain. Among the eight laminates, the highest flexural stress of 143 MPa was exhibited by laminate H, which had an outer layer of ramie fibre and core of flax fibre due to good bonding of matrix and reinforcement by vacuum infusion process. This hybrid laminate continued to absorb the load gradually increasing its flexural stress till it attained the value mentioned above. After that, the flexural stress decreased slowly before it failed at a strain value of 7.4%. The trend in the flexural stress-strain curve shows that this hybrid laminate exhibited ductile behaviour. Furthermore, all the test specimens failed in a ductile manner in all cases.

### 3.3. Compressive Strength

**Figure 10:**
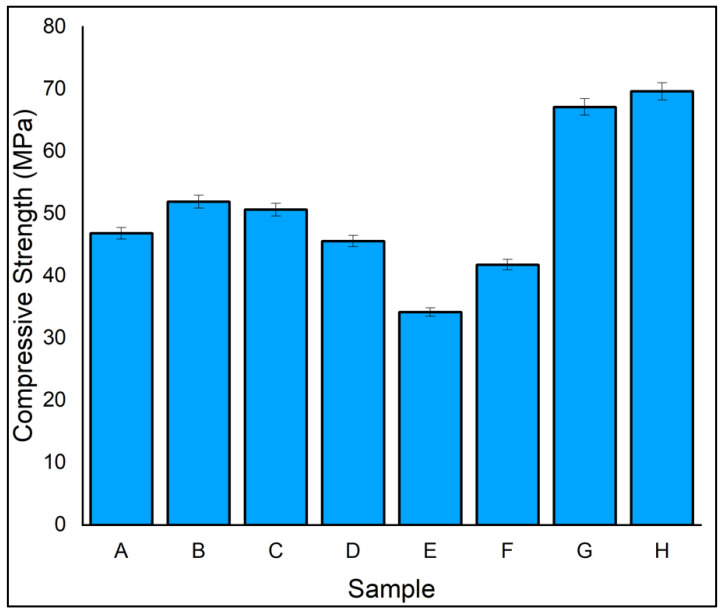
Compressive strength of specimen.

The matrix material has an important influence on the compressive strength of laminates. The length of the compressive test specimen is designed to eliminate global buckling and to develop pure compressive stress at the gauge section. In Figure 10, the compressive strength of laminate H with ramie as the outer layer and flax as the core is 69.65 MPa, the highest among the fabricated laminates. On the other hand, the compressive strength of laminate E with alternate layers of flax and ramie is 34.20 MPa, the lowest of the eight laminates. At the outset, the ability of the hybrid laminates to absorb the compressive force improved. Laminate B, having all five layers of ramie fibre, had 51.95 MPa, which was a 9.86% higher compressive strength than its counterpart, which had all five layers of flax fibres. A good fibre resistance to breakout is exhibited when ramie is placed as the outer layer [51]. The compressive strength plotted for the eight laminates showed a trend similar to that of the tensile test. It is inferred that the compressive behaviour of the laminates matched with the tensile test.

### 3.4. Impact Strength

**Figure 11:**
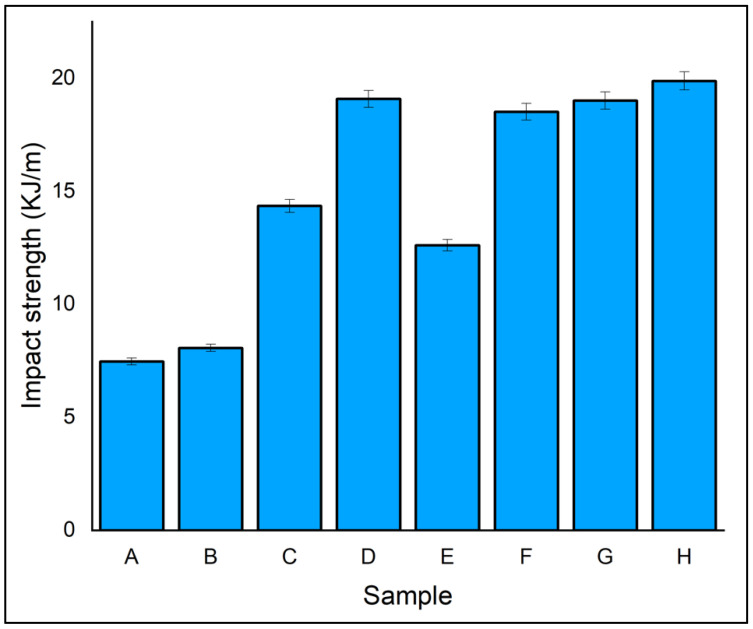
Impact strength of specimen.

Impact energy is the energy-absorbing capability of the material when it exerts a sudden load. The characteristics of matrix and fibre materials, the orientation of fibres, and the interfacial bonding of matrix and fibres influence the impact resistance of the composite laminates. A high strain rate is predicted in most real-time engineering applications [52]. The impact capability of the eight different composite laminates was tested using the Izod Charpy impact test. From Figure 11, it is evident that laminates A and B had lower impact strength than the hybrid laminates. Laminate A, with all five layers of flax fibres, had the lowest impact strength of all the fabricated laminates with 7.47 kJ. Laminate H had ramie as its outer layer and flax as its core. It had a high impact strength of 19.88 kJ, which was 36.57% higher than laminate E, since ramie fibre had better impact resistance than flax. The impact strength of the hybrid laminate increased due to the high interfacial strength of ramie fibre with phenol–formaldehyde resin applied to withstand impact loading [53,54,55,56]. Due to impact, the failure region showed complete separation of the laminate at the notch region. When adding ramie fibre to Kevlar/jute/banana fibre, the bonding strength of the fibres increased; hence, the impact strength also increased.

### 3.5. Hardness

**Figure 12:**
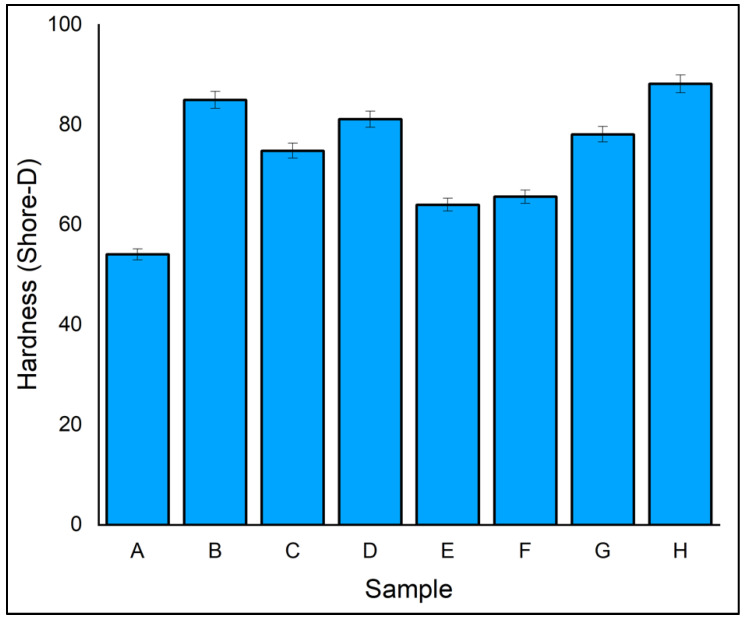
Hardness of specimen.

The hardness of a material is its capacity to withstand persistent deformation. Shore D hardness values were used to test the hardness of the fabricated laminates. The results are shown in Figure 12. Laminate A, which contained all five layers of flax, had a lower hardness value of 54 due to the low mechanical strength of flax fibre compared to ramie fibre. Laminate H showed the highest hardness value of 88.20 due to the presence and the strong bonding of ramie with flax fibre. Hybrid laminates C, D, E, F, G, and H showed higher hardness than laminate A. The results revealed that the incorporation of ramie fibre improved the hardness of the hybrid composite. Laminate H showed 27.40% higher hardness than laminate E, which may be due to the stacking of ramie and flax fibres. It is concluded that the laminates that have ramie fibre as skin material resist extra aberrations and penetration compared to laminates composed of flax fibre [57,58]. The increased stiffness of the flax and ramie fibres, in addition to the better interaction between the reinforcement fibres and phenol formaldehyde matrix, provide a high resistance to indentation by the indenter.

**Figure 13:**
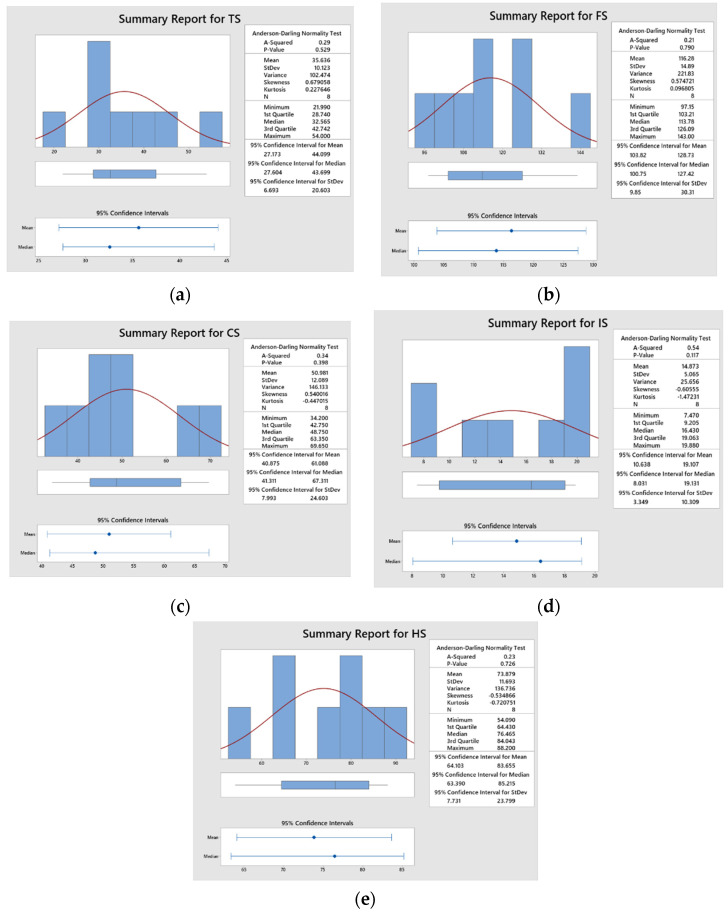
Statistical analysis of (**a**) tensile strength (**b**) flexural strength (**c**) compressive strength (**d**) impact strength (**e**) hardness.

Table 3 shows the experimental result of the composites. Figure 13 shows the statistical analysis of responses tensile, flexural, compressive, impact strength and hardness values obtained from Minitab 19 software (Minitab, LLC, USA) as presented below. It is observed that all the *p*-values are greater than 0.05, which shows that the measured experimental values were normally distributed.

### 3.6. Morphological Analysis

**Figure 14:**
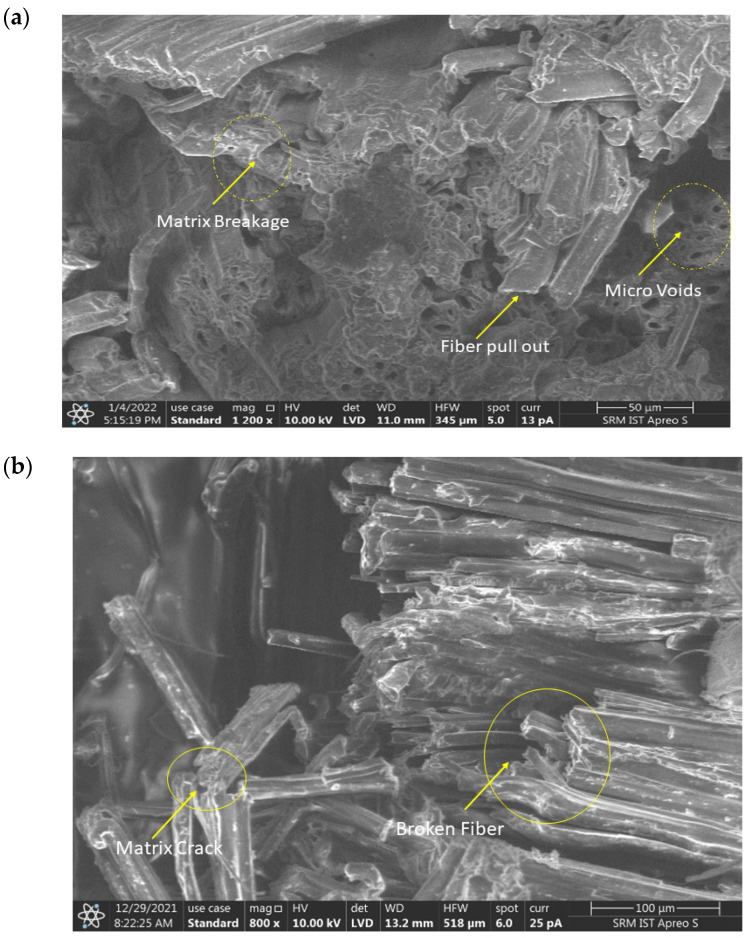

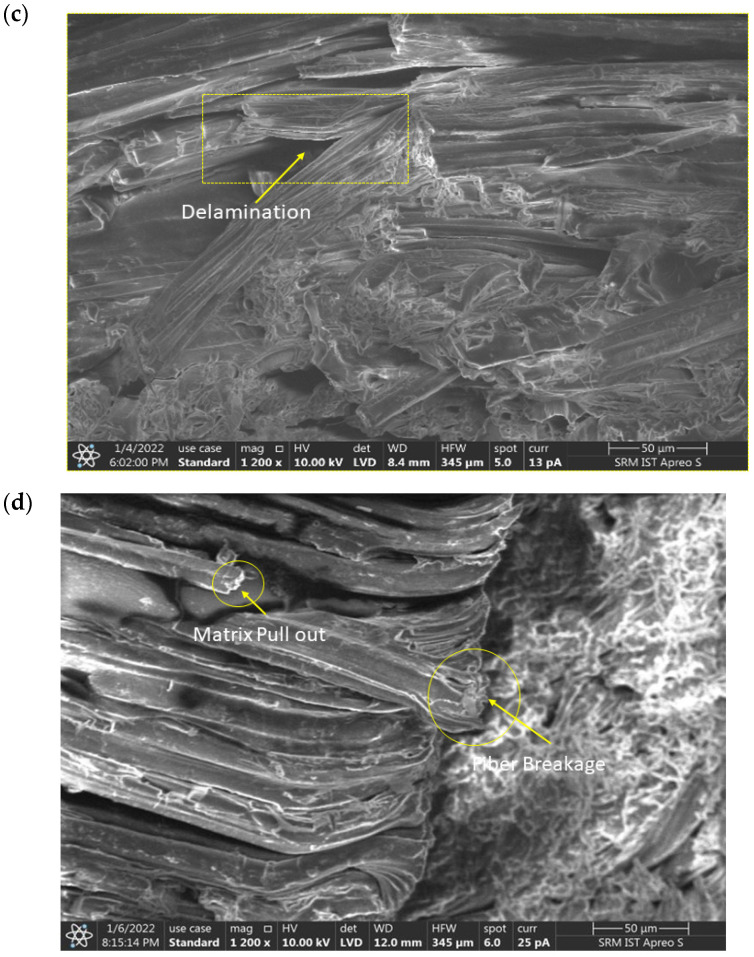
Morphological analysis of (**a**) tensile (**b**) flexural (**c**) compressive and (**d**) impact specimen.

The morphological analysis of ramie and flax fibre-reinforced hybrid composite at maximum condition was conducted using a scanning electron microscope. The good adhesion of fibre and resin was identified through the morphological analysis on laminate H when subjected to tensile loading. This is clearly illustrated in Figure 14a. Though the laminate was made using the vacuum infusion process there were noticeable defects such as micro voids. Due to these, some failures might have occurred. A detailed examination of the failure zone of laminate H reveals that the eventual failure was due to fibre pull-out and matrix cracking. The fibre reinforcement and the matrix were found to have good bonding. This allowed the laminate to absorb a sufficient load before failure [59,60].

The flexural test provided a detailed examination of the failure zone of laminate E. The flexural test reveals that the eventual failure was due to matrix cracking. The SEM from the flexural test specimen showed that matrix material continued to hold the reinforcement fibres. However, the matrix was pulled to its breaking point before undergoing brittle fracture [61]. Figure 14b clearly states that voids were found to be minimum due to the uniform load applied to the specimen.

Figure 14c shows the morphological analysis of laminate G when subjected to a compression test. Defects such as delamination and kinking of fibres were observed after the compression test. The reinforcement fibres underwent compression that led to embrittlement of the matrix material. The fibres that emerged from the matrix element were twisted and deformed under the influence of the compressive force. The matrix material exhibited both ductile and brittle fracture characteristics.

Figure 14d shows a morphological analysis of laminate B. The failure occurred due to a sudden impact load, resulting in fibre pull-out, matrix failure, and fibre breakage. Although the matrix had fragmented under the impact force, the fibres remained intact. These defects formed as the result of sudden application of the load during the impact test [62].

## 4. Conclusions

In this work, the development and evaluation of the hybridization effect on bidirectional flax fibre with ramie fibre reinforced with phenol–formaldehyde polymer composite fabricated through a vacuum infusion process have been studied experimentally under ASTM standards. The outcomes of this study are listed below.

- Laminate H had a better tensile strength of 54 MPa and better flexural strength of 143 MPa when compared with all other laminates. The main root cause was the interfacial adhesion of ramie and flax fibres with phenol–formaldehyde. The secondary reason for obtaining the better value was the process capability of the vacuum infusion method.
- The best compressive strength value of 69.65 MPa was observed in laminate H rather than in other laminates. The reason behind this was the use of ramie fibres as outer layers, which were used to absorb the applied compressive load.
- The hybrid laminate made with the casing of ramie fibres and the inter-core with flax fibres absorbed maximum impact energy of 19.88 kJ compared with its counterparts.
- The maximum hardness value obtained for laminate H was 88.2 from the Shore D hardness scale. Furthermore, it was observed that all the laminates had higher hardness values except the laminate that had all five layers of flax fibre.
- A detailed examination of the tensile, flexural, and impact failure zone of laminates was performed through morphological study. The combination of fibre breakage, fibre pull-out, and matrix cracking contributed to the eventual failure. The delamination and kinking of fibres were the main causes of the compressive failure of the laminates. In the vacuum infusion process, voids were reduced, and manufacturing defect was reduced. There was good adhesion of matrix and reinforcement, which improved the mechanical properties.

From the above inferences, it was concluded that the laminates with ramie fibre as the outer layer with the core of flax fibre exhibited better mechanical properties such as tensile, compressive, flexural and impact strength, and hardness value. The novelty of this proposed work is to develop the composites that deliver better mechanical properties than commercial composites available on the market, which can be used in lightweight structural applications. The present work can be extended by adding filler materials with natural fibres to broaden the application of polymer composites in various fields.

## Figures and Tables

**Table 1:** Physical properties of the reinforcement materials.

Physical Properties	Flax Fibre	Ramie Fibre
Density (g/cm^3^)	1.50	1.56
Tensile strength (MPa)	800	1000
Young’s modulus (GPa)	27.6	61.4–128
Elongation to break (%)	2.7–3.2	3.6–3.8

**Table 2:** Sequence of prepared specimen.

Sample	Specimen *	Weight of Laminate (g)	Thickness of Flax Fiber (mm)	Thickness of Ramie Fiber (mm)	Weight of Fiber (g)	Weight of Matrix (g)	Weight of Fiber (%)	Weight of Matrix (%)
A	FFFFF	412	4.15	-	131	281	32	68
B	RRRRR	374	-	4.60	124	250	33	67
C	FFRFF	364	3.32	0.92	128	236	35	65
D	RRFRR	396	0.83	3.68	127	269	32	68
E	FRFRF	352	2.49	1.84	129	223	37	63
F	RFRFR	374	1.66	2.76	129	245	35	65
G	FRRRF	358	1.66	2.76	129	229	36	64
H	RFFFR	372	2.49	1.84	130	242	35	65

* F—Flax: R—Ramie.

**Table 3:** Experimental result of Composite.

Sample	Specimen	Tensile Strength (MPa)	Flexural Strength (MPa)	Compressive Strength (MPa)	Impact Strength (KJ/m)	Hardness
A	FFFFF	30.99	114.95	46.85	7.47	54.09
B	RRRRR	33.00	125.3	51.95	8.07	85.01
C	FFRFF	32.13	109.85	50.65	14.35	74.82
D	RRFRR	27.99	112.6	45.6	19.08	81.14
E	FRFRF	21.99	97.15	34.2	12.61	64.03
F	RFRFR	42.99	101	41.8	18.51	65.63
G	FRRRF	42.00	126.35	67.15	19.01	78.11
H	RFFFR	54.00	143	69.65	19.88	88.2

F—Flax, R—Ramie.

## Data Availability

The data presented in this study are available through email upon request to the corresponding author.

## References

[1] Xian G., Guo R., Li C., Wang Y. (2022). Mechanical performance evolution and life prediction of prestressed cfrp plate exposed to hygrothermal and freeze-thaw environments. Compos. Struct..

[2] Kalita K., Mallick P.K., Bhoi A.K., Ghadai K.R. (2018). Optimizing Drilling Induced Delamination in GFRP Composites using Genetic Algorithm & Particle Swarm Optimisation. Adv. Compos. Lett..

[3] Behera R.R., Ghadai R.K., Kalita K., Banerjee S. (2016). Simultaneous prediction of delamination and surface roughness in drilling GFRP composite using ANN. Int. J. Plast. Technol..

[4] Mouritz A.P., Feih S., Kandare E., Mathys Z., Gibson A.G., Jardin P.E.D., Case S.W., Lattimer B.Y. (2009). Review of Fire Structural Modelling of Polymer Composites. Compos. Part A Appl. Sci. Manuf..

[5] Dong K., Hu K., Gao W. (2016). Fire Behavior of Full-Scale CFRP-Strengthened RC Beams Protected with Different Insulation Systems. J. Asian Arch. Build. Eng..

[6] Li C., Xian G. (2019). Experimental and Modeling Study of the Evolution of Mechanical Properties of PAN-Based Carbon Fibers at Elevated Temperatures. Materials.

[7] Chen Z., He X., Ge J., Fan G., Zhang L., Parvez A.M., Wang G. (2022). Controllable fabrication of nanofibrillated cellulose supported HKUST-1 hierarchically porous membranes for highly efficient removal of formaldehyde in air. Ind. Crops Prod..

[8] Wang Z., Zhao X.L., Xian G., Wu G., Raman R.K.S., Al-Saadi S. (2018). Effect of Sustained Load and Seawater and Sea Sand Concrete Environment on Durability of Basalt- and Glass-Fibre Reinforced Polymer (B/GFRP) Bars. Corros. Sci..

[9] Fiore V., Calabrese L. (2021). Effect of Glass Fiber Hybridization on the Durability in Salt-Fog Environment of Pinned Flax Composites. Polymers.

[10] Mochane M.J., Mokhena T.C., Mokhothu T.H., Mtibe A., Sadiku E.R., Ray S.S., Ibrahim I.D., Daramola O.O. (2019). Recent Progress on Natural Fiber Hybrid Composites for Advanced Applications: A Review. Express Polym. Lett..

[11] Kabir M.M., Wang H., Lau K.T., Cardona F. (2012). Chemical Treatments on Plant-Based Natural Fibre Reinforced Polymer Composites: An Overview. Compos. B Eng..

[12] Lubis M.A.R., Handika S.O., Sari R.K., Iswanto A.H., Antov P., Kristak L., Lee S.H., Pizzi A. (2022). Modification of Ramie Fiber via Impregnation with Low Viscosity Bio-Polyurethane Resins Derived from Lignin. Polymers.

[13] Moghtadernejad S., Barjasteh E., Johnson Z., Stolpe T., Banuelos J. (2021). Effect of thermo-oxidative aging on surface characteristics of benzoxazine and epoxy copolymer. J. Appl. Polym. Sci..

[14] He L., Li W., Chen D., Zhou D., Lu G., Yuan J. (2015). Effects of Amino Silicone Oil Modification on Properties of Ramie Fiber and Ramie Fiber/Polypropylene Composites. Mater. Eng..

[15] Aristri M.A., Lubis M.A.R., Laksana R.P.B., Sari R.K., Iswanto A.H., Kristak L., Antov P., Pizzi A. (2022). Thermal and Mechanical Performance of Ramie Fibers Modified with Polyurethane Resins Derived from Acacia Mangium Bark Tannin. J. Mater. Res. Technol..

[16] Liu Z.-T., Yang Y., Zhang L., Liu Z.W., Xiong H. (2007). Study on the Cationic Modification and Dyeing of Ramie Fiber. Cellulose.

[17] Pandey J.K., Ahn S.H., Lee C.S., Mohanty A.K., Misra M. (2010). Recent Advances in the Application of Natural Fibre Based Composites. Macromol. Mater. Eng..

[18] Ku H., Wang H., Pattarachaiyakoop N., Trada M. (2011). A review on the tensile properties of natural fibre reinforced polymer composites. Compos. Part B Eng..

[19] Peças P., Carvalho H., Salman H., Leite M. (2018). Natural fibre composites and their applications: A review. J. Compos. Sci..

[20] Luo G., Xie J., Liu J., Zhang Q., Luo Y., Li M., Jiang Z. (2023). Highly conductive, stretchable, durable, breathable electrodes based on electrospun polyurethane mats superficially decorated with carbon nanotubes for multifunctional wearable electronics. Chem. Eng. J..

[21] Naveen J., Jawaid M., Zainudin E.S., Sultan M.T.H., Yahaya R. (2019). Mechanical and Moisture Diffusion Behaviour of Hybrid Kevlar/Cocos Nucifera Sheath Reinforced Epoxy Composites. J. Mater. Res. Technol..

[22] Giridharan R. (2019). Preparation and property evaluation of Glass/Ramie fibres reinforced epoxy hybrid composites. Compos. Part B Eng..

[23] Zhang Y., Wen B., Cao L., Li X., Zhang J. (2015). Preparation and properties of unmodified ramie fibre reinforced polypropylene composites. J. Wuhan Univ. Technol.-Mater. Sci. Ed..

[24] Torres-Arellano M., Renteria-Rodríguez V., Franco-Urquiza E. (2020). Mechanical Properties of Natural-Fibre-Reinforced Biobased Epoxy Resins Manufactured by Resin Infusion Process. Polymers.

[25] Li W., Krehl J., Gillespie J.W., Heider D., Endrulat M., Hochrein K., Dubois C.J. (2004). Process and Performance Evaluation of the Vacuum-Assisted Process. J. Compos. Mater..

[26] Hsiao K.-T., Heider D. (2012). Vacuum assisted resin transfer molding (VARTM) in polymer matrix composites. Manufacturing Techniques for Polymer Matrix Composites (PMCs).

[27] Cicala G., Pergolizzi E., Piscopo F., Carbone D., Recca G. (2018). Hybrid composites manufactured by resin infusion with a fully recyclable bioepoxy resin. Compos. Part B Eng..

[28] Sanjeevi S., Shanmugam V., Kumar S., Ganesan V., Sas G., Johnson D.J., Das O. (2021). Effects of water absorption on the mechanical properties of hybrid natural fibre/phenol formaldehyde composites. Sci. Rep..

[29] Dilfi K.F.A., Che Z.J., Xian G.J. (2019). Grafting of Nano-Silica onto Ramie Fiber for Enhanced Mechanical and Interfacial Properties of Ramie/Epoxy Composite. J. Zhejiang Univ. Sci. A.

[30] Chen M., Lu Z. (2015). Load Transfer Mechanism of the Composites Incorporating Nanohybrid Shish-Kebab Structures. Compos. Struct..

[31] Swamy R.P., Kumar G.C.M., Vrushabhendrappa Y., Joseph V. (2004). Study of Areca-Reinforced Phenol Formaldehyde Composites. J. Reinf. Plast. Compos..

[32] Thakur V.K., Singha A.S. (2010). Mechanical and Water Absorption Properties of Natural Fibres/Polymer Biocom-posites. Polym. -Plast. Technol. Eng..

[33] Joseph S., Sreekala M.S., Oommen Z., Koshy P., Thomas S. (2002). A comparison of the mechanical properties of phenol formaldehyde composites reinforced with banana fibres and glass fibres. Compos. Sci. Technol..

[34] Sathyaseelan P., Sellamuthu P., Palanimuthu L. (2020). Influence of stacking sequence on mechanical properties of areca-kenaf fibre-reinforced polymer hybrid composite. J. Nat. Fibres.

[35] Dunne R., Desai D., Sadiku R., Jayaramudu J. (2016). A review of natural fibres, their sustainability and automotive applications. J. Reinf. Plast. Compos..

[36] Lotfi A., Li H., Dao D.V., Prusty G. (2019). Natural fibre-reinforced composites: A review on material, manufacturing, and machinability. J. Thermoplast. Compos. Mater..

[37] Sujon M.A.S., Habib M.A., Abedin M.Z. (2020). Experimental investigation of the mechanical and water absorption properties on fibre stacking sequence and orientation of jute/carbon epoxy hybrid composites. J. Mater. Res. Technol..

[38] EL-Wazery M.S., El-Kelity A.M.E., Elsad R.A. (2020). Effect of Water Absorption on the Tensile Characteristics of Natural/Synthetic Fabrics Reinforced Hybrid Composites. Int. J. Eng..

[39] Da Silva R.V., Voltz H., Filho A.I., Milagre M.X., Machado C.D.C. (2020). Hybrid composites with glass fibre and natural fibres of sisal, coir, and luffa sponge. J. Compos. Mater..

[40] Fabris H.J., Knauss W.G. (1989). Comprehensive Polymer Science and Supplements. Synthetic Polymer Adhesives.

[41] Roy A., Naskar A., Ghosh A., Adhikari J., Saha P., Ghosh M. (2021). Hybrid Plastics and Natural Materials. Reference Module in Materials Science and Materials Engineering.

[42] Raja T., Ravi S., Karthick A., Afzal A., Saleh B., Arunkumar M., Prasath S. (2021). Comparative Study of Mechanical Properties and Thermal Stability on Banyan/Ramie Fibre-Reinforced Hybrid Polymer Composite. Adv. Mater. Sci. Eng..

[43] Mohanavel V., Raja T., Yadav A., Ravichandran M., Winczek J. (2021). Evaluation of Mechanical and Thermal Properties of Jute and Ramie Reinforced Epoxy-based Hybrid Composites. J. Nat. Fibres.

[44] Bajpai P.K., Singh I., Madaan J. (2014). Development and Characterization of PLA-Based Green Composites: A Review. J. Thermoplast. Compos. Mater..

[45] Gomes A., Matsuo T., Goda K., Ohgi J. (2007). Development and Effect of Alkali Treatment on Tensile Properties of Curaua Fiber Green Composites. Compos. Part A Appl. Sci. Manuf..

[46] He L.P., Tian Y., Wang L.L. (2008). Study on Ramie Fibre Reinforced Polypropylene Composites (RF-PP) and its Mechanical Properties. Adv. Mater. Res..

[47] Ashok D., Puhan S., Pradhan R., Babu P.K., Reddy Y.S. (2020). An Experimental Investigation of New Hybrid Composite Material Using Ramie-Flax and Its Mechanical Properties through Finite Element Method. Recent Trends Mech. Eng..

[48] Gupta S., Haq M.I.U., Mohan S., Anand A., Raina A., Dutta V., Kumar R. (2019). Evaluation of mechanical properties of ramie/banana reinforced hybrid composites. J. Mech. Eng. (JMechE).

[49] Yousif B.F., Shalwan A., Chin C.W., Ming K.C. (2012). Flexural Properties of Treated and Untreated Kenaf/Epoxy Composites. Mater. Eng..

[50] Vedanarayanan V., Kumar B.S.P., Karuna M.S., Jayanthi A., Kumar K.V.P., Radha A., Christopher D. (2022). Experimental Investigation on Mechanical Behaviour of Kevlar and Ramie Fibre Reinforced Epoxy Composites. J. Nanomater..

[51] Herrera-Franco P.J., Valadez-González A. (2005). A Study of the Mechanical Properties of Short Natural-Fiber Reinforced Composites. Compos. B Eng..

[52] Biswas S., Kindo S., Patnaik A. (2011). Effect of fibre length on mechanical behavior of coir fibre reinforced epoxy composites. Fibres Polym..

[53] Kumar R., Anand A. (2019). Fabrication and mechanical characterization of Indian ramie reinforced polymer compo-sites. Mater. Res. Express.

[54] Almeida Pontes L.D., Netto P.A., Ferreira J.B., Margem F.M., Monteiro S.N. (2016). Flexural Mechanical Character-ization of Polyester Composites Reinforced with Ramie Fibres. Charact. Miner. Met. Mater..

[55] Kapila K., Samanta S., Kirtania S. (2021). Fabrication and Characterization of Ramie Fibre Based Hybrid Composites. Recent Advances in Mechanical Engineering.

[56] Ramesh M., Rajeshkumar L., Balaji D. (2021). Mechanical and Dynamic Properties of Ramie Fibre-Reinforced Composites. Mech. Dyn. Prop. Biocomposites.

[57] Srinivasan V.S., Boopathy S.R., Sangeetha D., Ramnath B.V. (2014). Evaluation of Mechanical and Thermal Properties of Banana–Flax Based Natural Fibre Composite. Mater. Eng..

[58] Sarwar A., Mahboob Z., Zdero R., Bougherara H. (2020). Mechanical Characterization of a New Kevlar/Flax/Epoxy Hybrid Composite in a Sandwich Structure. Polym. Test..

[59] Haameem J.A.M., Majid M.S.A., Afendi M., Marzuki H.F.A., Fahmi I., Gibson A.G. (2016). Mechanical Properties of Napier Grass Fibre/Polyester Composites. Compos. Struct..

[60] Özturk S. (2010). Effect of Fiber Loading on the Mechanical Properties of Kenaf and Fiberfrax Fiber-Reinforced Phenol-Formaldehyde Composites. J. Compos. Mater..

[61] Chaudhary V., Bajpai P.K., Maheshwari S. (2018). Studies on Mechanical and Morphological Characterization of Developed Jute/Hemp/Flax Reinforced Hybrid Composites for Structural Applications. J. Nat. Fibers.

[62] Zhong J.B., Lv J., Wei C. (2007). Mechanical properties of sisal fibre reinforced Ureaformaldehyde resin composites. Express Polym. Lett..

